# Imported taeniasis in the context of colon hydrotherapy

**DOI:** 10.1002/ccr3.7717

**Published:** 2023-07-23

**Authors:** Bárbara Gomila, Verónica Pedrón‐García, David Granizo‐Bermejo, J. Guillermo Esteban

**Affiliations:** ^1^ Microbiology Service General University Hospital of Castellón Castellón Spain; ^2^ Línea Natural® Valencia Spain; ^3^ Advanced Institute for Holistic Health Madrid Spain; ^4^ Department of Parasitology, Faculty of Pharmacy University of Valencia Valencia Spain

**Keywords:** colon hydrotherapy, proglottids, *Taenia saginata*

## Abstract

We report a case of imported taeniasis, under the modality of “visiting friends and relatives”, in the context of colon hydrotherapy. This technique allows the detection of proglottids, diagnosed in this case as *Taenia saginata* based on the gravid proglottid that presented more than 13 uterine branches and showed active motility. Moreover, the patient did not consume pork for religious reasons. The treatment with paromomicin sulfate was effective. In this case, a trip to Ethiopia, together with the ingestion of raw beef, was the cause of parasitization. It is highly advisable to obtain detailed information from the patient on their background, especially their travel and dietary histories.

## Graphical abstract

(A) Vision of the sediment of the formaldehyde/ethyl acetate technique, observing different *Taenia eggs*; (B) *Taenia egg* showing the unrecognizability of the oncosphere hooks (interdifferential contrast microscopy); (C) *Taenia egg* with the outer primarymembrane; (D) Gravid proglottid of *Taenia* rinsed and mounted with Amann's Lactophenol between two slides.

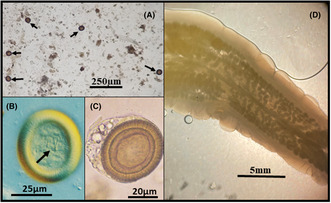

## 1 INTRODUCTION

Modern Colon Hydrotherapy® is the most advanced technique to recover normal physiological peristalsis and cleanse the large intestine in its entirety. It is a technique that consists of the deep irrigation of the entire large intestine with filtered and purified water, through a cannula that is introduced into the rectum. This cannula is two‐way, that is, water enters one way (irrigation tube), and fecal matter leaves through another tube (evacuation tube) several times during the session, without the need of having to evacuate in a toilet.^1^ Colon hydrotherapy achieves three fundamental objectives: the stimulation of the enteric plexuses of the colon, the recovery and strengthening of the intestinal muscles, and the cleansing of the intestinal lumen. This technique is convenient, hygienic, painless, and odorless, while being at the same time a gentle and safe therapy without any harmful or undesirable effects.^1^

By means of this technique, an imported case of taeniasis (the human parasitization by representatives of the genus *Taenia*) was diagnosed in Valencia (Spain), under the modality of “visiting friends and relatives.” There are three human species whose intermediate hosts are pigs (*Taenia solium* and *Taenia asiatica*) and cattle (*Taenia saginata*), so that humans become infected by ingesting raw or undercooked pork or beef harboring the infective or cysticercus form.^2^

## 2 CASE REPORT

**FIGURE 1:**
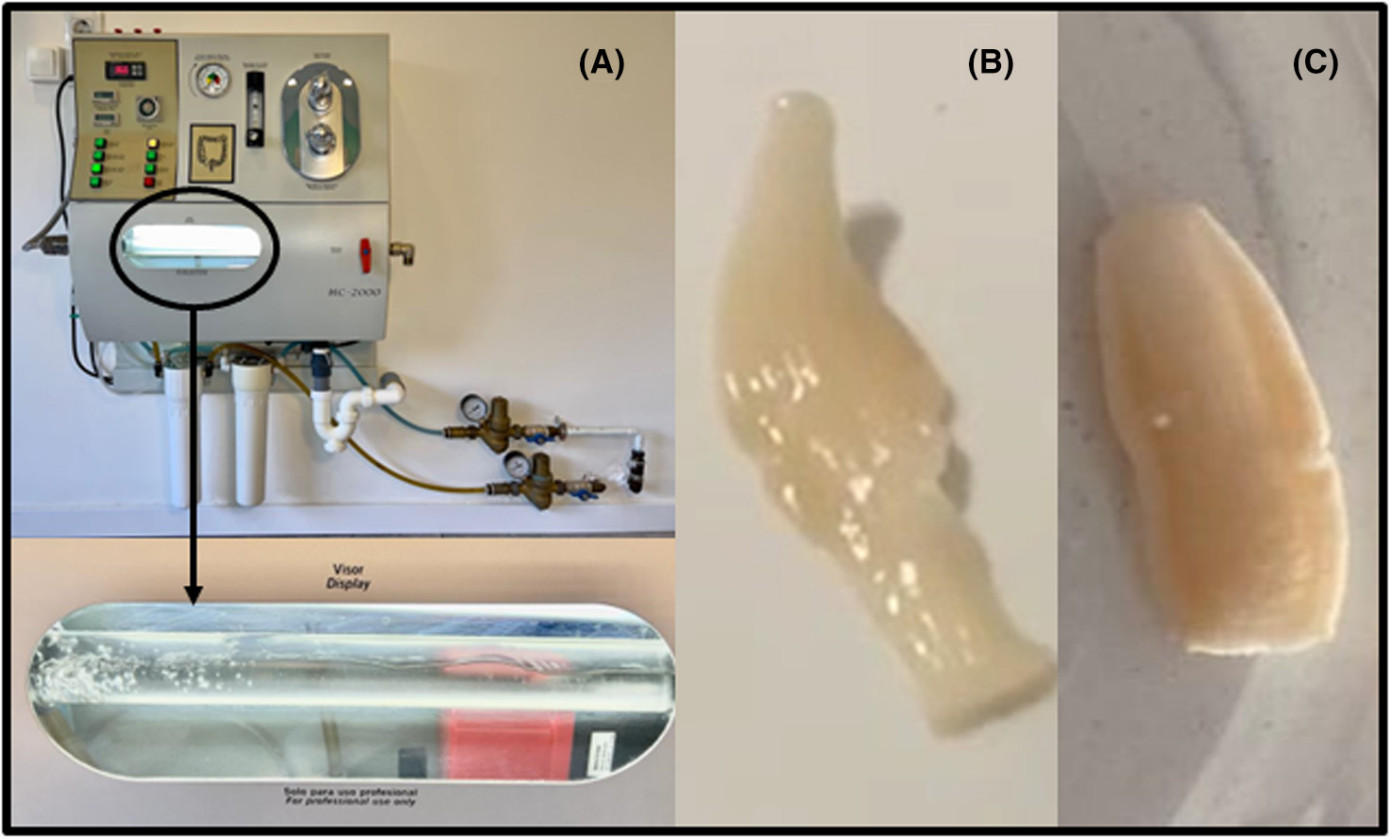
(A) Apparatus used in colon hydrotherapy showing the display to examine the contents of the colon; (B, C) Proglottids eliminated by the patient: mobile (B) and fixed (C)

**FIGURE 2:**
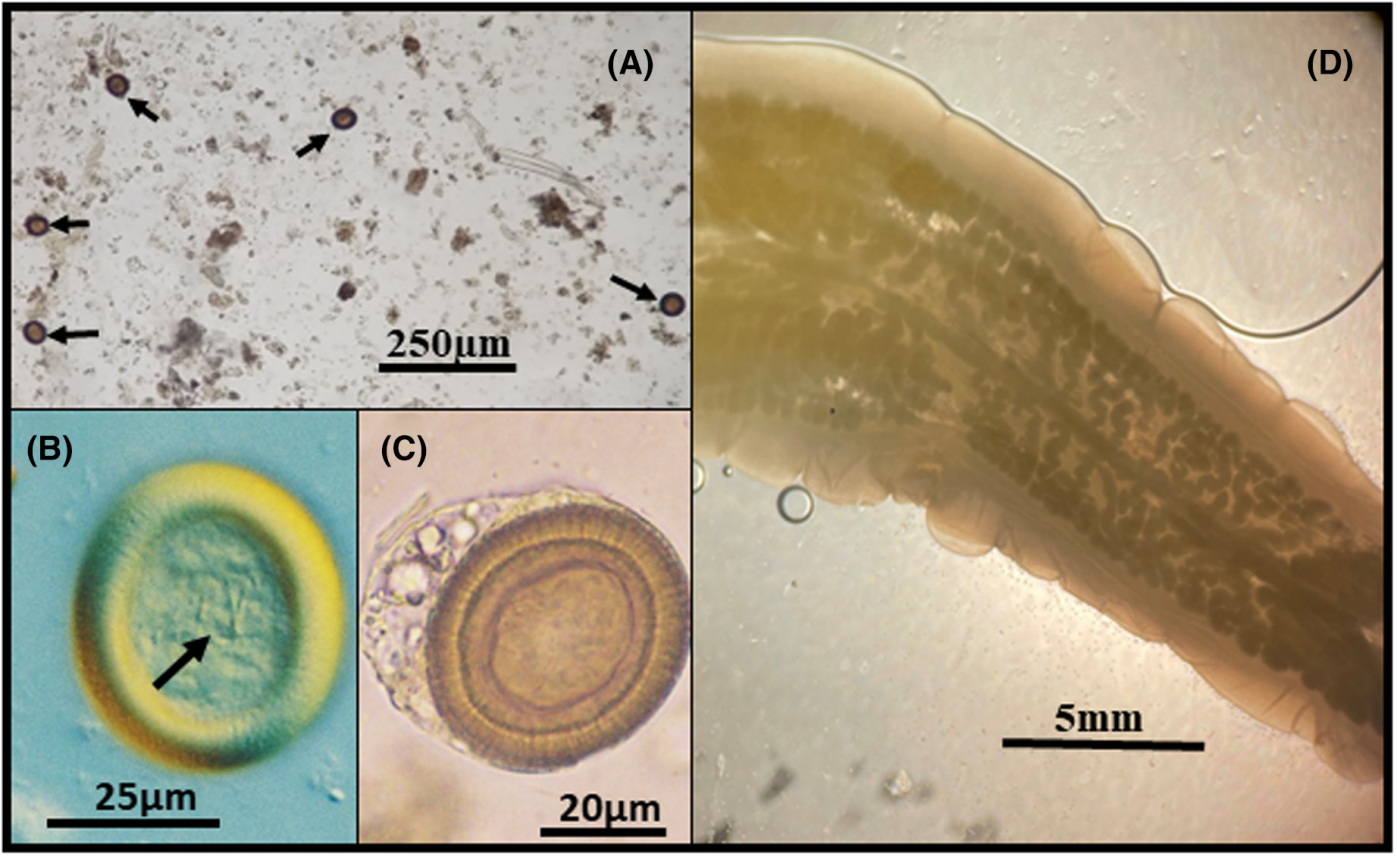
(A) Vision of the sediment of the formaldehyde/ethyl acetate technique, observing different *Taenia* eggs (scale: 250 μm); (B) *Taenia* egg showing the unrecognizability of the oncosphere hooks (interdifferential contrast microscopy; scale: 25 μm); (C) *Taenia* egg with the outer primary membrane (scale: 20 μm); (D) Gravid proglottid of *Taenia* rinsed and mounted with Amann's Lactophenol between two slides (scale: 5 mm)

In Valencia in March 2022, a 35‐year‐old patient, born in Dese (Ethiopia), and residing in Spain since he was 13 years old, begins to feel unwell while presenting asthenia, abdominal pain, heavy digestion, and flatulence. Initially, he takes a natural product obtained from a herbalist. Since the symptoms do not subside, he decides to have colon hydrotherapy (Figure 1A). The device used in this technique contains a display through which the therapist can observe some whitish structures. The patient, subsequently, is able to observe in the toilet bowl some moving whitish structures (Figure 1B). Next, the analysis of three fecal samples is performed, which are all negative. Faced with this situation, the therapist calls the Microbiology Service of the General University Hospital of Castellón. Subsequently, the Department of Parasitology of the Faculty of Pharmacy of the University of Valencia was called to proceed with the examination and diagnosis of the aforementioned structures (Figure 1C). Three serialized fecal samples and three Graham tapes obtained on consecutive days were analyzed. Macroscopic analysis of the whitish structures pointed to the presence of *Taenia* proglottids. The analysis of the feces, through the formaldehyde/ethyl acetate concentration technique, revealed the presence of eggs (Figure 2A) of spherical morphology, yellow‐brownish color, 32.5–40.0 μm in diameter, with a thick radially fluted cover, being evident when observed under a microscope (Figure 2B,C). In addition, the egg contained an embryo with six hooks (= oncosphere), discernible with difficulty (Figure 2B), and which can easily be confused with some pollen (e.g., *Ligustrum*) with a resemblance in size, color, and appearance. Sometimes, a thin outer primary membrane can be seen around some eggs, especially when the proglottid breaks (Figure 2C). All this confirmed the presence of *Taenia* eggs. Graham tapes presented negative results. The analysis of one of the proglottids with Amann's Lactophenol showed the presence of a lateral genital pore, as well as the existence of more than 13 primary lateral branches on each side of the central uterine axis (Figure 2D). This allowed the diagnosis of *T. saginata/T. asiatica*, both presenting the same morphological characteristics and whose differentiation is made through molecular diagnosis.^3^, ^4^ However, in the present case it suggested parasitization by *T. saginata* since the gravid ring had more than 13 uterine branches, showing active motility, and the patient's non‐consumption of pork for religious reasons. Paromomycin sulfate (Humatin®), in oral doses of 50 mg/kg, divided into 3 doses/day, was prescribed. Fecal parasitological tests carried out in May and June after treatment were negative.

## 3 DISCUSSION

Colon hydrotherapy is a technique that has nothing to do with laxatives or enemas. In addition, it is particularly recommended in digestive, dermatological, immune, metabolic, neuropsychiatric, vascular, and genitourinary disorders; likewise, it is highly being especially recommended in the prevention of rectal and colon cancer. However, this technique does not allow the elimination of infections caused by the different etiological agents.^1^

When talking about intestinal parasites, the parasitic structures that occur in the feces (trophozoites, cysts, oocysts, spores, eggs, and larvae) are smaller than 250 μm, except in the case of adult helminths, in which they may be larger than 1 cm (Cestoda: *Diphyllobothrium, Hymenolepis, Dipylidium, Taenia*; Nematoda: *Enterobius, Trichuris*). In these cases, they could be seen by the therapist in the display of the colon hydrotherapy device, a situation that occurred in the present case.

The trip made to Ethiopia by the patient, in December 2021 to see his family, was relevant when establishing the epidemiological chain of the case. Ethiopia is a country in sub‐Saharan Africa where most households have direct contact with domestic animals, resulting in high human taeniasis and bovine cysticercosis infection prevalences.^5^ In addition, eating raw beef dishes, such as “kurt” or “kitfo”, allows the transmission of this zoonotic disease.^6^ Moreover, the patient belongs to the Muslim community and does not eat pork.

At symptomatological level, most patients do not usually present any symptoms, despite the great deal of literature on intestinal and extraintestinal complications related to *T. saginata*.^7^, ^8^ In any case, the patient presented an abdominal symptomatology without specific parasitic characterization.

The drugs of choice for the treatment of taeniasis are praziquantel (5‐10 mg/kg single‐dose) and niclosamide (50 mg/kg single‐dose, up to a maximum of 2 g).^9^ As both drugs are not marketed in Spain, paromomycin sulfate was prescribed.^10^ In Ethiopia, niclosamide and praziquantel are very common drugs to treat this zoonosis, although mebedanzole and albendazole are also used.^11^ What is surprising is the number of people who resort to traditional herbal treatments, whose efficacy and risk are unknown, as is the case with the flowers of the rosacea *Hagenica abyssinia* (“Kosso/Heto”), containing a toxin, closely related to folic acid, and the cause of some cases of liver carcinoma and blindness.^6^, ^12^

Poor sanitation, ingestion of undercooked infected meat, a lack of education in general and inadequate health education in particular as well as the scarce availability of tenicides are the main obstacles in the control of this disease. Therefore, public health education especially with regard to an increased cooking time of meat is required. Furthermore, freezing meat at −20°C for at least 24 h would be sufficient, at least in industrialized countries, to prevent this cestodiasis.

## 4 CONCLUSION

We highlight a typical case of “visiting friends and relatives,” where the trip to Ethiopia, together with the ingestion of raw beef, was the cause of the parasitization by *T. saginata*. Consequently, it is highly advisable to obtain from the patient detailed information on their background, especially their travel and dietary histories, which should allow an adequate diagnosis.

## AUTHOR CONTRIBUTIONS

**Bárbara Gomila:** Conceptualization; formal analysis; methodology. **Verónica Pedrón‐García:** Data curation; methodology. **David Granizo‐Bermejo:** Data curation; methodology. **J. Guillermo Esteban:** Conceptualization; formal analysis; investigation; validation; visualization; writing – original draft; writing – review and editing.

## FUNDING INFORMATION

None.

## CONFLICT OF INTEREST STATEMENT

The authors declare no conflict of interest.

## CONSENT STATEMENT

Written informed consent was obtained from the patient to publish this report in accordance with the journal's patient consent policy.

## Data Availability

Data openly available in a public repository that issues datasets with DOIs.
